# The Emergence of Language in the Hominin Lineage: Perspectives from Fossil Endocasts

**DOI:** 10.3389/fnhum.2017.00427

**Published:** 2017-08-23

**Authors:** Amélie Beaudet

**Affiliations:** ^1^School of Geography, Archaeology and Environmental Studies, University of the Witwatersrand Johannesburg, South Africa; ^2^Department of Anatomy, University of Pretoria Pretoria, South Africa

**Keywords:** paleoneurology, endocasts, *Australopithecus*, sulcal patterns, Broca's area

## From endocasts to brains

**Figure 1:**
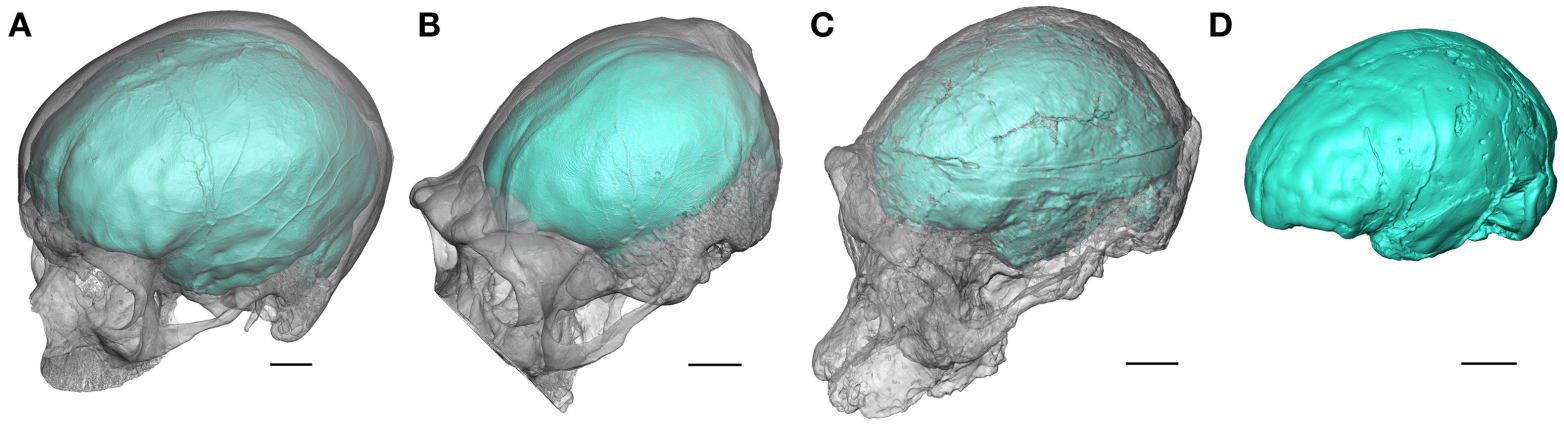
Oblique views of the endocasts of an extant human **(A)**, an extant chimpanzee **(B)**, and of the *Australopithecus africanus* specimens Sts 5 **(C)**, and Sts 60 **(D)** from Sterkfontein (South Africa). Sts 60 is a natural endocast associated to the fragmented cranium TM 1511. The braincase is rendered semi-transparent. Scale bars: 2 cm.

Since brain does not fossilize, brain endocast (i.e., replica of the inner surface of the braincase, Figure 1) constitutes the only direct evidence for reconstructing hominin brain evolution (Holloway, 1978; Holloway et al., 2004a). In this context, paleoneurology has suffered from strong limitations due to the fragmentary nature of the fossil record and the absence of any information regarding subcortical elements in extinct taxa. Additionally, variation in brain shape and organization (and in the corresponding endocast) is technically difficult to capture, as stated by Bruner (2017a, p. 64): “[…] the smooth and blurred geometry of the brain, its complex and complicated mechanisms, and its noticeable individual variability make any research associated with its morphology very entangled and difficult to develop within fixed methodological approaches.” An emblematic example might be the reluctance of paleoneurologists to consider the sulcal imprints visible on the endocranial surface because of the substantial uncertainties in describing such features in fossil specimens and related debates (e.g., the lunate sulcus in the Taung child's endocast; Falk, 1980a, 2009, 2014; Holloway, 1981a; Holloway et al., 2004b). In 1987, Tobias even came to the conclusion that “The recognition of specific cerebral gyri and sulci from their impressions on an endocast is a taxing, often subjective and even invidious undertaking which arouses much argumentation” (p. 748). However, in conjunction with a conceptual shift toward a more comprehensive overview of hominin brain evolution (e.g., reconsideration of the “cerebral rubicon” characterizing the human brain, Falk, 1980b; Holloway, 1983), continuous discoveries of new fossil material and recent analytical developments are progressively improving and refining our knowledge about the human neural evolutionary history. In particular, paleoneurology is producing new evidence for reconstructing the timing and mode of the emergence of crucial functions, such as language.

## What fossil endocasts can tell us: cortical organization and shape

When the neurocranium is filled with sediment during the fossilization process, information about brain morphology and organization may be recorded as a natural endocast (e.g., Figure 1D). For fossil specimens not preserving a natural endocast, it is possible to generate a virtual imprint of the endocranial surface (Figures 1A–C). Within the limitations discussed above, the endocast provides evidence for tracking both structural and morphological changes in brain within the hominin lineage (Holloway, 1978; Holloway et al., 2004a; Zollikofer and Ponce de León, 2013). In this context, fossil endocasts can potentially deliver critical data for identifying cerebral reorganization with implications for speech capacity. In particular, the frontal lobe is involved in crucial functions suggested to be specific to extant humans. Among them, language has been invariably linked to the Broca's cap configuration and the lateral aspect of the frontal lobes has been consequently largely explored in the hominin fossil record. The human-like configuration of the Broca's area has long been acknowledged to emerge concomitantly with the genus *Homo* (Falk, 1983; Tobias, 1987; see also the earliest descriptions of the Broca's cap in fossil humans, e.g., Kappers, 1929; Connolly, 1950). However, besides being largely questioned in the extant primate taxa (rev. in Bruner, 2017b), recent reinvestigation of the fossil record has revealed a more complex history of the reorganization of the frontal lobes, and more particularly of the inferior frontal gyrus and Broca's cap, deeply rooted in the early representatives of the hominin lineage.

In terms of structural organization, the identification of two differential patterns in the extant ape and human frontal lobes, characterized, respectively, by the presence of the fronto-orbital sulcus and of the horizontal and ascending branches of the lateral fissure, was previously considered as an effective trait for identifying the derived condition of the Broca's cap in the fossil record (rev. in Falk, 2014). In extant humans, even if the correspondence between the sulcal pattern and cytoarchitectonic areas is questioned (Amunts et al., 1999), the two rami of the lateral fissure delineate the Brodmann's areas 44 and 45 involved in language production and comprehension (Falk, 2014). Within this framework, while the ape-like pattern was described in the South African *Australopithecus* hypodigm, the endocasts of the earliest human representatives were suggested to be closer to the extant human condition (Falk, 1983, 2014; Tobias, 1987). However, this structural feature turned out to be more complex than previously thought. In particular, the high-resolution virtual investigation of the *Australopithecus sediba* endocast (MH 1) adds further complexity to this purported dichotomy since this specimen combines a chimpanzee-like sulcal pattern with evidence of shape reorganization (Carlson et al., 2011). Similarly, Holloway et al. (2004a) thoroughly explored the fossil hominin record and reported occurrences of shape frontal asymmetries at the level of the Broca's cap in early hominins (e.g., Sts 5), confirming that this configuration is not confined to *Homo* (Tobias, 1987). A leftward asymmetry of the Broca's cap is commonly reported in extant humans and considered to be functionally relevant for speech capacities, even if still largely debated (rev. in Keller et al., 2009). Imaging techniques and 3D modeling of the virtual endocast supported the assumption of the presence of frontal asymmetries in non-human fossil hominins by mapping shape asymmetries (Braga and Thackeray, 2007; Beaudet et al., 2014). Moreover, landmark-based analyses of the asymmetries of the third frontal convolution in fossil and extant hominids demonstrated a gradual increase in the degree of expression of asymmetries in the human lineage and emphasize the need of not only describing the occurrence but also quantifying the “magnitude” of endocranial asymmetries (Balzeau et al., 2014).

Given that the latest studies challenged our previous thoughts on the emergence of the human-like configuration of the frontal lobes (see also Beaudet and Bruner, in press), the next step would be to reconsider the available evidence from a new perspective, notably by overcoming current limitations in paleoneurology. The development of new imaging techniques, such as phase contrast X-ray synchrotron microtomography (e.g., Carlson et al., 2011) or neutron microtomography (e.g., Beaudet et al., 2016a; Zanolli et al., 2017), pushes further the practical limits inherent to the non-invasive investigation of endostructural features in fossil specimens. The major difficulty in discussing the sulcal pattern in endocasts, and the configuration of critical cortical areas such as the Broca's cap, lies in the absence of reliable protocols for automatically identifying the furrows on the fossil endocranial surface, as currently performed on the MRIs of extant human brains (e.g., Fischer et al., 2012). However, recent efforts in applying computer-assisted approaches for automatic sulci detection in fossil primate endocasts have paved the way for future methodological developments (Beaudet et al., 2016b). Similarly, the successful application of landmark-free deformation-based models for exploring fossil primate endocranial surfaces (e.g., Durrleman et al., 2012; Beaudet et al., 2016b; Beaudet and Bruner, in press) and assessing endocranial asymmetries (Kitchell, 2017) are providing encouraging perspectives for applications in paleoneurology. As a concrete example of future applications, since the extant human Broca's cap is characterized by a particular morphoarchitecture (i.e., combination of specific sulcal organization and pattern of shape asymmetries of the inferior frontal gyrus), the construction of a statistical model (e.g., atlas) reporting 3D variations of the sulcal pattern (as previously performed in 2D by Connolly, 1950) and external morphology of this area in extant hominids would allow to automatically characterize the fossil condition and test critical hypothesis (e.g., reorganization of the inferior frontal gyrus in *Australopithecus*). By integrating the intra-specific morphological variability and provide objective data, such a protocol would represent a valuable tentative for overcoming serious limitations in paleoneurology. Consequently, we might predict that fresh evidence will certainly emerge from the systematic reinvestigation of the fossil record with the assistance of imaging techniques and 3D modeling methods.

## Looking for further evidence

Speech capacity cannot be appropriately inferred only from the cerebral condition, therefore hypotheses aiming at reconstructing the timing and mode of emergence of language in the hominin lineage should seek to combine various lines of evidence. The endocast is the only direct proxy for reconstructing the fossil neural condition and cerebral capacities in extinct species. However, additional cranial regions might contribute to understand the development of language capacity in the human evolution. As for instance, the petrous bone is frequently preserved in the fossil record and imaging techniques allow non-invasive access to the bony labyrinth and thus, to the hearing system (Spoor, 1993). Quantitative analysis of early hominin specimens revealed that changes are observed in the proportions of the inner ear (notably the oval window and cochlear length) in the hominin lineage that might be related to an increase in low-frequency sensitivity in extant humans as compared to early hominins (Braga et al., 2015). Similarly, the middle ear ossicles (malleus, incus and stapes) can be virtually investigated by the means of high-resolution imaging techniques in fossil hominin taxa for predicting auditory sensitivity levels of the hearing range, and more specifically in the lower frequencies (Stoessel et al., 2016). Interestingly, the audibility of specific frequencies is suggested to impact speech perception (e.g., high-frequencies, Stelmachowicz et al., 2004). In this context, because of the relationship between shape and functions in specific components of the inner ear (e.g., cochlea; Manoussaki et al., 2008), one may imagine possible associations between reconstruction of fossil auditory capabilities and language development.

Additionally, through the identification of genes related to language capacity in extinct hominin species (e.g., FOXP2; Krause et al., 2007) and the description of bones directly involved in speech production in the fossil record (e.g., hyoid bones; Arensburg et al., 1989, 1990), both genetic and anatomical studies undoubtedly constitute invaluable sources of information for evaluating the ability to speak of extinct species (rev. in Lieberman, 2006, 2007). Besides the hyoid bone, the length of the neck and the cranial-base angle are critical for reconstructing the fossil hominin vocal tract (Lieberman, 2007). Interestingly, these structures are preserved in the early hominin fossil record, as illustrated by the recent description of the entire cervical column of the *A. afarensis* specimen DIK-1-1 (Ward et al., 2017) or the studies of the basicranial shape and flexion in the most complete *A. africanus* cranium Sts 5 (“Mrs Ples”) (e.g., Ross and Henneberg, 1995; Spoor, 1997), and may contribute to the discussion of the emergence of language.

Similarly, cultural, technical and social contexts certainly played a critical role in the development of language capacities. Indeed, experiments dealing with tool making and functional MRIs revealed the complex pattern of neuronal stimuli in such activities and the intimate relationship with language (Stout et al., 2008), as anticipated by paleoneurological studies (Holloway, 1969, 1981b). Furthermore, these experiments emphasize the importance of learning processes in stone tool manufacture and support the potential influence of inter-individual interactions in the emergence of language (“social brain hypothesis”; Dunbar, 1993). Finally, life history traits should also be considered when searching for the origins of language, particularly since brain plasticity has been revealed to play an important role in the emergence of the human behavior and cognitive traits, including language (rev. in Gómez-Robles and Sherwood, 2016).

Accordingly, a comprehensive approach both in terms of practical investigation (i.e., combining quantification of variation in organization and shape of the endocranial surface) and theoretical background (i.e., integrating biological, social and cultural hypotheses) is essential and unavoidable for understanding the context of the emergence of language. As a whole, research in paleoneurology would significantly benefit from a “holistic” approach to the evolution of the human brain.

## Author contributions

The author confirms being the sole contributor of this work and approved it for publication.

### Conflict of interest statement

The author declares that the research was conducted in the absence of any commercial or financial relationships that could be construed as a potential conflict of interest.
